# Supplementary material for: Effect of Posttranslational Modifications on the Structure and Activity of FTO Demethylase
Source: Int J Mol Sci. 2021 Apr 26;22(9):4512. doi: 10.3390/ijms22094512 (PMC8123419; doi:10.3390/ijms22094512)
Supplement: Supplementary file 1 [file ijms-22-04512-s001.zip › ijms-1161488-supplementary.pdf]

International Journal of *Molecular Sciences*

MDPI

Article

Supplementary Material

Effect of posttranslational modifications on the structure and activity of FTO demethylase.

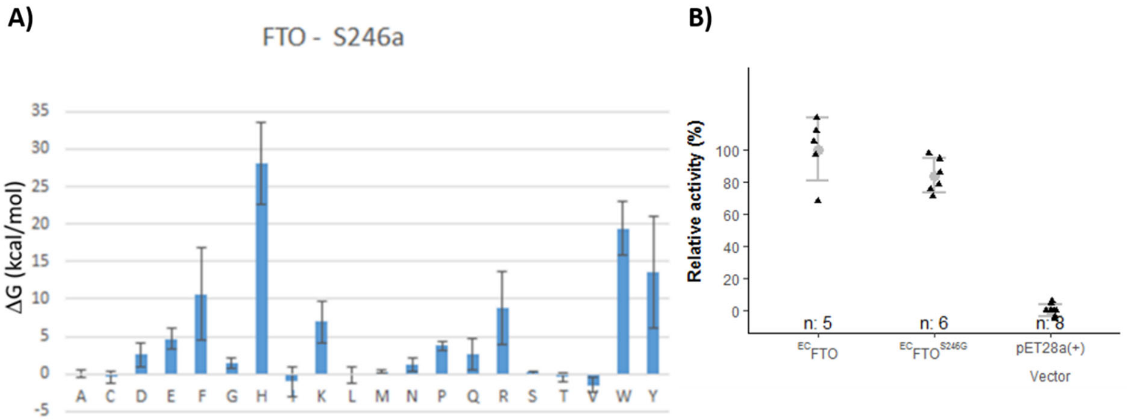

Supplementary Figure 1. ***In silico*** **and** ***in vitro*** **S246G substitution effect on FTO parameters. (A)** Prediction of the impact of various S246 amino acid substitutions on FTO protein stability. The effect of the S246G substitution was estimated at 0.5 kcal/mol, which is little in comparison to other substitutions. **(B)** Enzymatic activity of ^{EC}FTO^{S246G} mutant based on chemiluminescence assay. N^6-meA was used as a substrate. Relative activity is referenced to the activity of 1μg of ^{EC}FTO (100%). Data are represented as points and means, with whiskers showing sample standard deviation. n – number of samples.

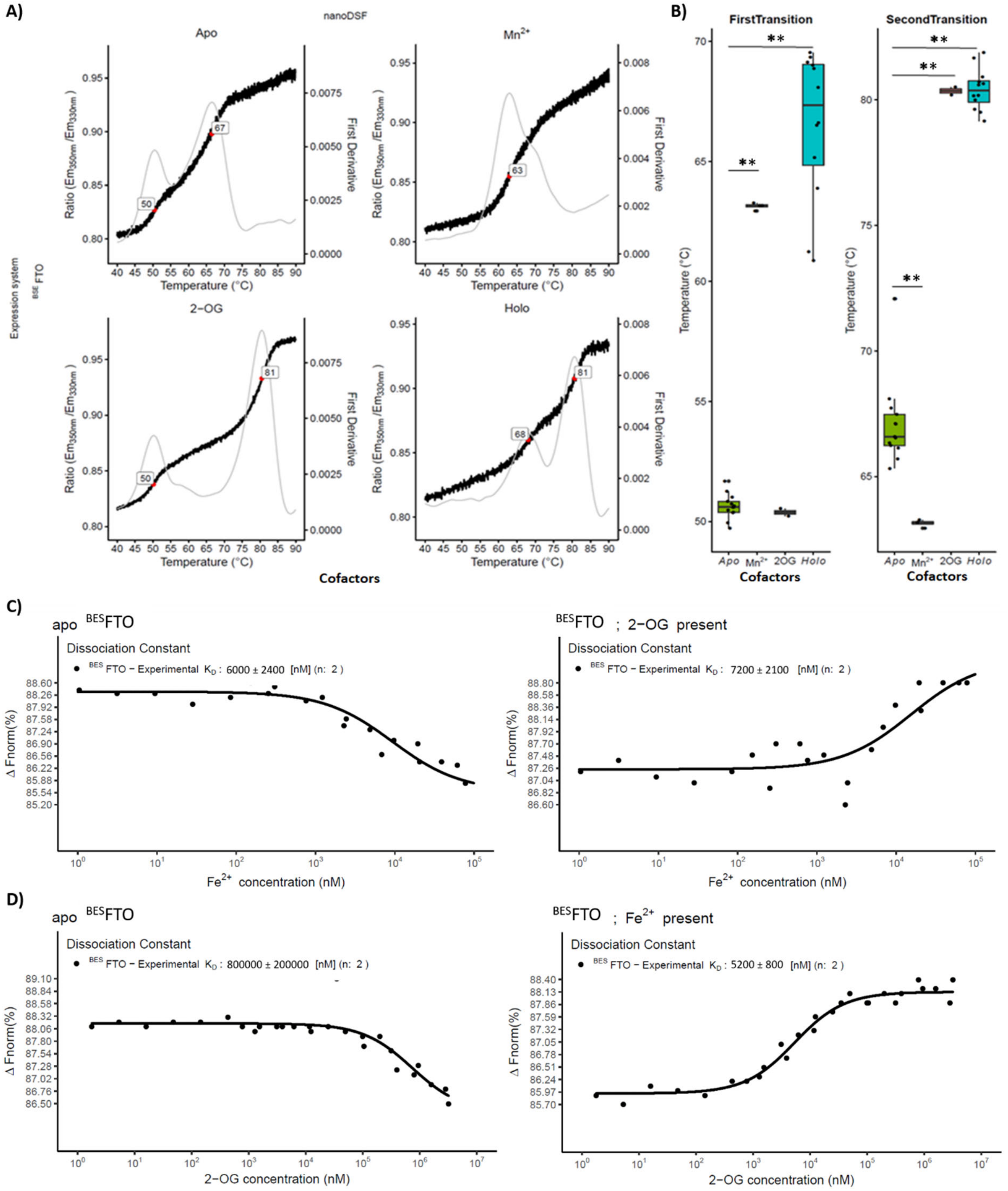

Supplementary Figure 2. Effect of cofactors on ^{BES}FTO parameters (A,B). NanoDSF analysis of the ^{BES}FTO thermal stability in the absence (apo) or presence of Mn^{2+}, 2-OG, or both cofactors (holo). A) Ratio of emissions (bold lines) and first derivative (thin lines) curves are shown as a function of temperature. Under all conditions, except the Mn^{2+} alone, two unfolding transitions (red dots) occurs: the first one (T_{m1}) less prominent, at a lower temperature, and the second one (T_{m2}) more clear, at a higher temperature. The first transition is affected by Mn^{2+}, while the second one changes in the presence of 2-OG. (B) Comparison of ^{BES}FTO melting temperatures under different experimental conditions. Gathered data are represented as a boxplots. Box represents the interquartile range. Whiskers show the highest and the lowest data points. Horizontal line within a box represents median value. n – number of samples. ** $P < 0.05$. **(C,D)** MST analysis of ^{BES}FTO interaction with Fe^{2+} **(C)** and 2-OG **(D)**. The plot represents dose response curves (changes of the initial fluorescence during thermophoresis) of labeled

^{BES}FTO in the presence of the increasing concentrations of the one ligand in the presence (right) or abscence (left) of the second. Under both conditions ^{BES}FTO interacts with Fe^{2+}, with similar K_D. The ^{BES}FTO-2-OG complex in the Fe^{2+} absence showed ~150 times higher K_D than in Fe^{2+} presence indicating that Fe^{2+} presence was crucial for ^{BES}FTO-2-OG interaction. K_D are presented as estimated value ± standard deviation. n –number of repetitions.

Amino acid	Position	^{EC}FTO		^{BES}FTO	
		Amount of peptides carrying modification (score)	Amount of peptides without modification	Amount of peptides carrying modification (score)	Amount of peptides without modification
T	6	1	3	3	26
T	32	0	29	2	125
Y	39	0	32	1	188
S	55	0	56	3	541
S	56	1	55	5	525
T	105	0	35	1	196
Y	106	1	34	0	197
Y	108	0	1	1	135
T	111	0	1	1	35
T	138	2	104	0	57
S	173	0	43	1	169
S	183	0	99	8	657
S	184	0	99	**23**	**643**
Y	185	0	99	6	661
S	195	0	0	1	814
Y	214	0	3	2	201
S	229	1	196	2	331
S	256	0	1	**49**	**81**
S	260	0	1	**30**	**123**
S	313	0	39	8	79
S	318	0	3	1	112
S	319	0	3	1	109
T	320	0	3	1	109
S	327	0	75	1	166
S	355	0	65	8	105
S	358	0	15	2	81
T	393	0	10	1	62
S	458	0	0	4	51
S	482	0	27	1	198
S	494	0	27	1	314

Supplementary Figure 3. MS analysis of putative phosphorylation sites in ^{EC}FTO and ^{BES}FTO. Tables show the amount of specific peptides carrying a modified residue in comparison to unmodified ones. Phosphorylations significantly exceeded the set threshold (>20 detected peptides with modification) are present only in ^{BES}FTO (in bold).

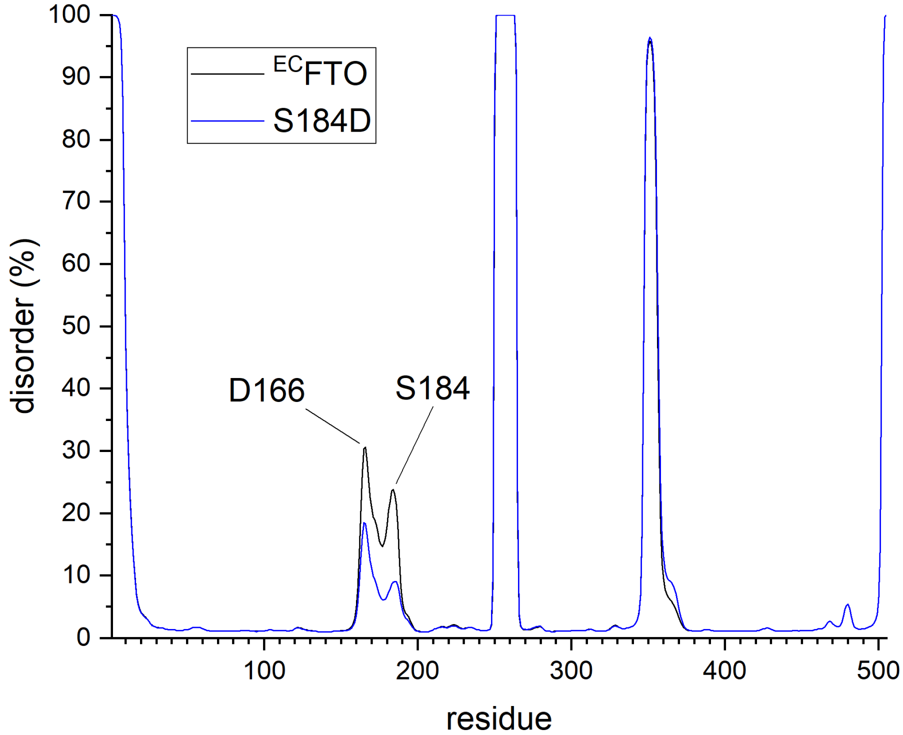

Supplementary Figure 4. Effect of S184D substitution on FTO stability. Profile of the protein disorder was estimated for unmodified FTO and S184D variant with RaporX server. The partially disordered loop (N164-G187), which is invisible in the PDB structures, is significantly stabilized by S184D replacement.

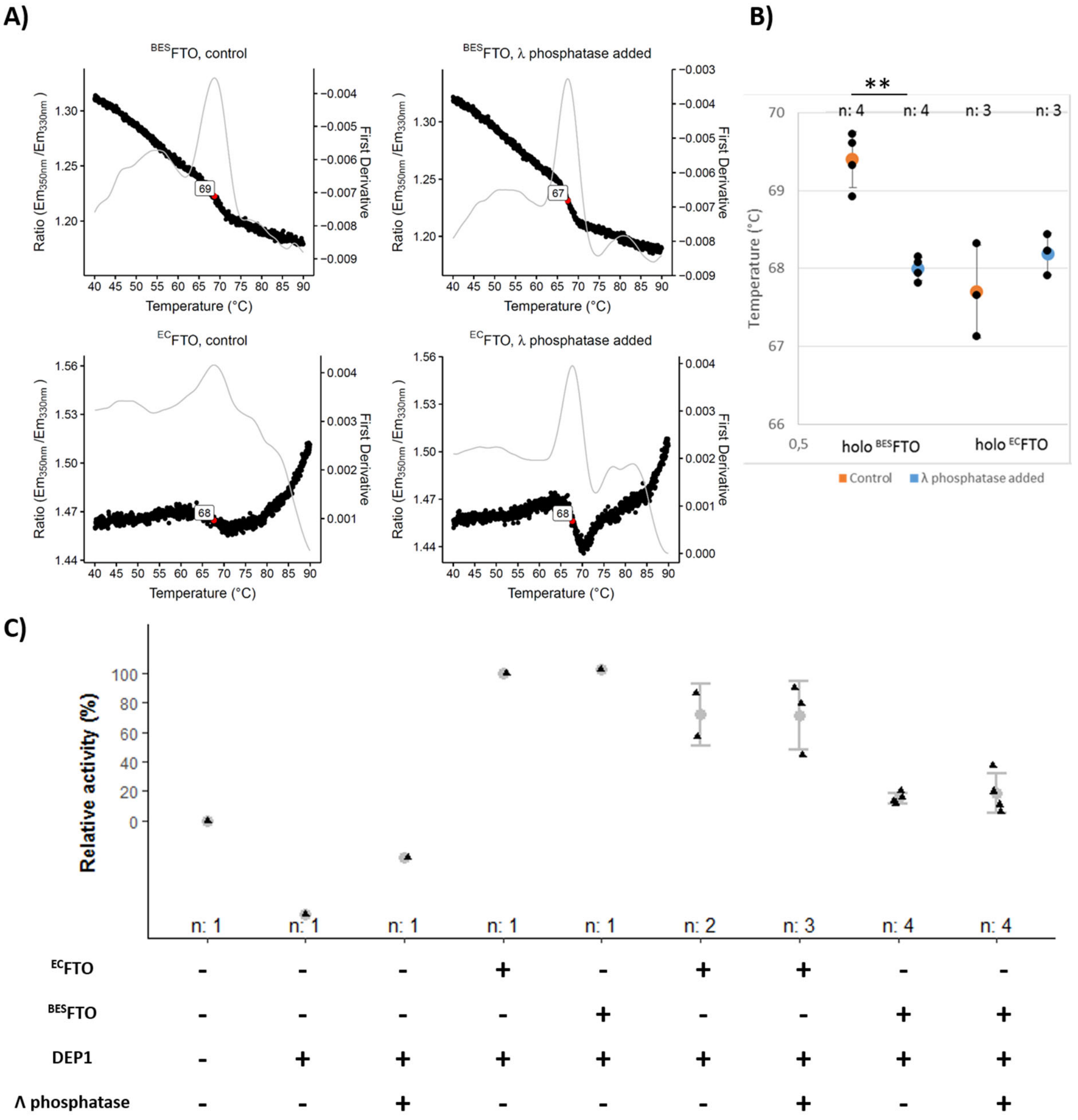

Supplementary Figure 5. Dephosphorylation effect on FTO parameters. A,B) NanoDSF analysis of the dephosphorylationeffect on the thermal stability of purified ^{EC}FTO and ^{BES}FTO in the presence of Mn^{2+} and 2-OG (holo). Unfolding temperatures of all preparations were determined in the presence of the Mn^{2+} and 2-OG after λ phosphatase treatment (blue) or through control experiment without enzyme (orange). For ^{BES}FTO, λ phosphatase significantly decreases the T_m. A) Plots illustrate ratio of emissions (bold lines) and first derivative (thin lines) curves as a function of temperature. All four protein preparations display one distinct unfolding transition (red dots). B) Gathered data are represented as points and means, with whiskers showing sample standard deviation. n – number of samples. ** $P < 0.05$. C) Effect of dephosphorylation procedure on ^{EC}FTO and ^{BES}FTO enzymatic activity. N^6-meA was used as substrate. Relative activity is referenced to activity of 1 µg ^{EC}FTO (100%).. Data are represented as points and a means with whiskers showing standard deviation. n – number of independent experiments.

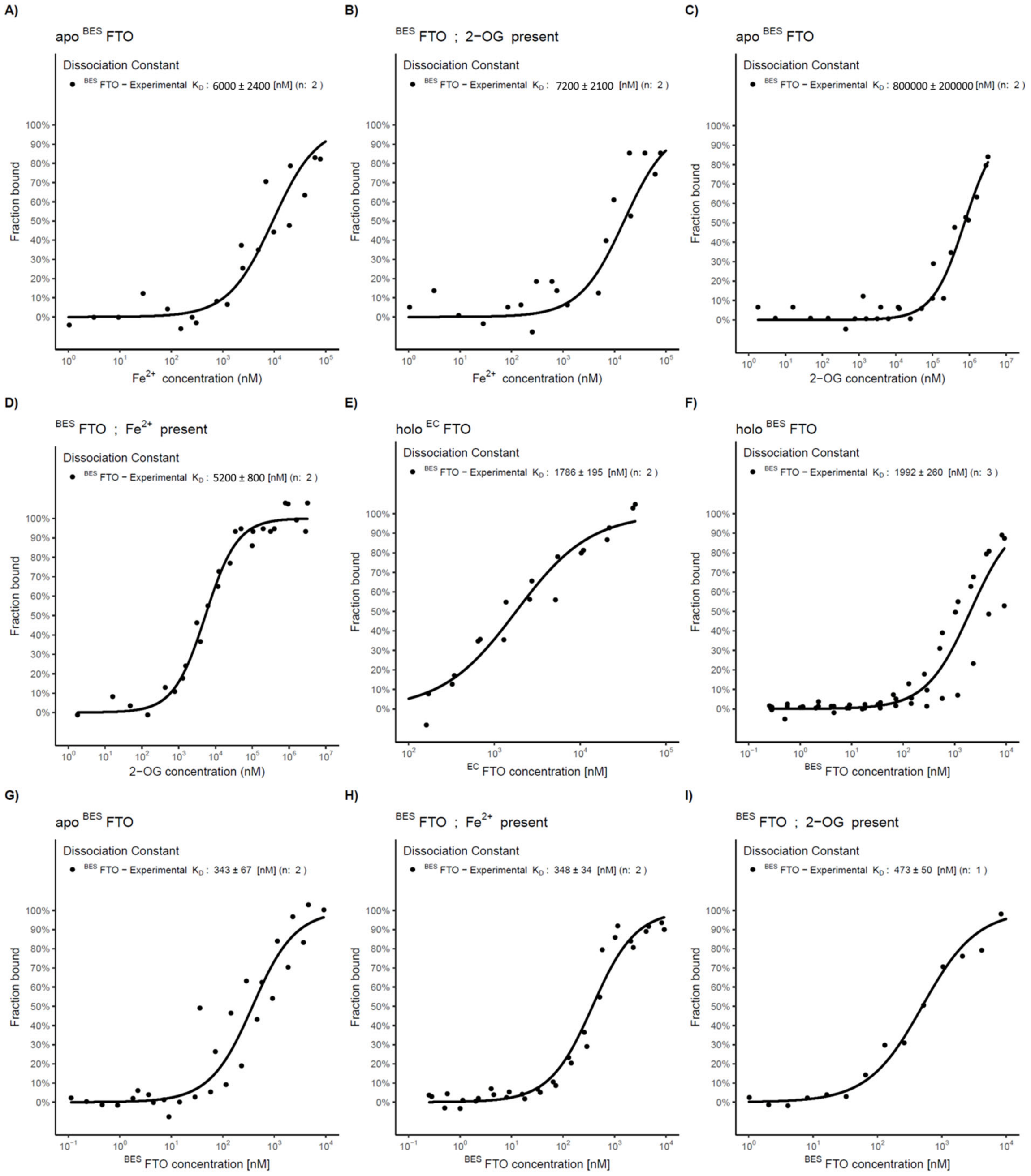

Supplementary Figure 6. Comparison of the MST experiments. (A-I). The plot represents complex level of labeled protein at a given FTO concentration for each separate sample (data points) and for modeled equilibrium between protein alone and the complex (straight lines). K_D are shown as estimated value ± standard deviation. n –number of repetitions.

Supplementary Table 1. PCR primers

FTO S246G (forward)	5′- CGGTGGCAGTGTAC**G**GTTATAGCTGTGAAG-3′
FTO S246G (reverse)	5′- CTTCACAGCTATAAC**C**GTACACTGCCACCG-3′
FTO S256D (forward)	5′- GCCCTGAAGAGGAA**GA**TGAGGATGACTCTC-3′
FTO S256D (reverse)	5′- GAGAGTCATCCTCA**TC**TTCCTCTTCAGGGC -3′
FTO construct (forward)	5′-AGCGGCTCTTCAATGAAGCGCACCCCGACTGCC-3′
FTO construct (reverse)	5′-AGCGGCTCTTCTCCCGGGTTTTGCTTCCAGAAGCT-3′

Amino acid sequences of recombinant FTO proteins. Amino acid sequence of His-Tag is underlined

^{EC}FTO:

MGSSHHHHHHSSGLVPRGSHMKRTPTAEEREREAKKLRLLEELEDTWLPYLTPKDDEFYQQ
WQLKYPKLILREASSVSEELHKEVQEAFLTLHKHGCLFRDLVRIQGKDLLTPVSRILIGNPGCT
YKYLNTRLFTVPWPVKGSNIKHTEAEIAAACETFLKLNDYLQIETIQALEELAAKEKANEDAV
PLCMSADFPRVGMGSSYNGQDEVDIKSRAAYNVTLLNFMDPQKMPYLKEEPYFGMGKMAV
SWHHDENLVDRSAVAVYSYSCEGPEEESEDDSHLEGRDPDIWHVGFKISWDIETPGLAIPLHQ
GDCYFMLDDLNATHQHCVLAGSQPRFSSTHRVAECSTGTLDYILQRCQLALQNVCDDVDND
DVSLKSFEPAVLKQGEEIHNEVEFEWLRQFWFQGNRYRKCTDWWCQPMAQLEALWKKMEG
VTNAVLHEVKREGLPVEQRNEILTAILASLTARQNLRREWHARCQSRIARTLPADQKPECRPY
WEKDDASMPLPFDLTDIVSELRGQLLEAKP

^{EC}FTO-S246G, ^{BES}FTO:

MGSSHHHHHHSSGLVPRGSHMKRTPTAEEREREAKKLRLLEELEDTWLPYLTPKDDEFYQQ
WQLKYPKLILREASSVSEELHKEVQEAFLTLHKHGCLFRDLVRIQGKDLLTPVSRILIGNPGCT
YKYLNTRLFTVPWPVKGSNIKHTEAEIAAACETFLKLNDYLQIETIQALEELAAKEKANEDAV
PLCMSADFPRVGMGSSYNGQDEVDIKSRAAYNVTLLNFMDPQKMPYLKEEPYFGMGKMAV
SWHHDENLVDRSAVAVYGYSCEGPEEESEDDSHLEGRDPDIWHVGFKISWDIETPGLAIPLHQ
GDCYFMLDDLNATHQHCVLAGSQPRFSSTHRVAECSTGTLDYILQRCQLALQNVCDDVDND
DVSLKSFEPAVLKQGEEIHNEVEFEWLRQFWFQGNRYRKCTDWWCQPMAQLEALWKKMEG
VTNAVLHEVKREGLPVEQRNEILTAILASLTARQNLRREWHARCQSRIARTLPADQKPECRPY
WEKDDASMPLPFDLTDIVSELRGQLLEAKP

^{EC}FTO-S256D:

MGSSHHHHHHSSGLVPRGSHMKRTPTAEEREREAKKLRLLEELEDTWLPYLTPKDDEFYQQ
WQLKYPKLILREASSVSEELHKEVQEAFLTLHKHGCLFRDLVRIQGKDLLTPVSRILIGNPGCT
YKYLNTRLFTVPWPVKGSNIKHTEAEIAAACETFLKLNDYLQIETIQALEELAAKEKANEDAV
PLCMSADFPRVGMGSSYNGQDEVDIKSRAAYNVTLLNFMDPQKMPYLKEEPYFGMGKMAV
SWHHDENLVDRSAVAVYSYSCEGPEEEDEDDSHLEGRDPDIWHVGFKISWDIETPGLAIPLHQ
GDCYFMLDDLNATHQHCVLAGSQPRFSSTHRVAECSTGTLDYILQRCQLALQNVCDDVDND
DVSLKSFEPAVLKQGEEIHNEVEFEWLRQFWFQGNRYRKCTDWWCQPMAQLEALWKKMEG
VTNAVLHEVKREGLPVEQRNEILTAILASLTARQNLRREWHARCQSRIARTLPADQKPECRPY
WEKDDASMPLPFDLTDIVSELRGQLLEAKP
